# Relationship between weekend catch-up sleep and the risk of diabetic kidney disease

**DOI:** 10.20945/2359-4292-2024-0370

**Published:** 2025-04-24

**Authors:** Xia Wu, Yunhai Tang, Yayun He, Zhihuan Tang, Yingdan Zhao

**Affiliations:** 1 Department of Nephrology, Jiading Branch of Shanghai General Hospital, Shanghai Jiao Tong University School of Medicine, Shanghai, P. R. China; 2 Department of Nephrology, Shanghai General Hospital, Shanghai, P. R. China

**Keywords:** Weekend catch-up sleep, diabetic kidney disease, estimated glomerular filtration rate, proteinuria, patients with diabetes

## Abstract

**Objective:**

To investigate the association between weekend catch-up sleep (WCS) and the risk of diabetic kidney disease (DKD). Subjects and

**methods:**

Data from 1,621 adults aged 18 years or older from the National Health and Nutrition Examination Survey 2017-2020 were obtained for this cross-sectional study. WCS was calculated as the mean weekend sleep duration minus the mean weekday sleep duration. The outcomes were DKD, a reduced estimated glomerular filtration rate (eGFR), and proteinuria. The associations between WCS and DKD, the reduced eGFR or proteinuria were evaluated via a weighted multivariate logistic regression model. Subgroup analyses were performed for different sexes and participants with or without hypertension.

**Results:**

A total of 583 diabetic patients had DKD, of whom 198 patients displayed reduced eGFRs and 499 patients had proteinuria. After adjusting for all confounding factors, Group 4 (weekend CUS ≥ 2 and < 3 hours) still had lower odds of DKD [odds ratio (OR) = 0.51, 95% confidence interval (CI): 0.28-0.93] and proteinuria (OR = 0.51, 95% CI: 0.27-0.96). Additionally, subgroup analyses stratified by sex and hypertension consistently revealed connections in female diabetic patients (OR = 0.40, 95% CI: 0.20-0.78 for DKD; OR = 0.47, 95% CI: 0.22-0.97 for proteinuria) and in diabetic patients with hypertension (OR = 0.39, 95% CI: 0.18-0.81 for DKD; OR = 0.38, 95% CI: 0.19-0.77 for proteinuria). However, the fully adjusted model revealed no such association between WCS and a reduced eGFR.

**Conclusion:**

WCS was found to decrease the likelihood of developing DKD and proteinuria among American adult patients diagnosed with diabetes, particularly among female patients or those with hypertension.

## INTRODUCTION

Diabetes poses a significant public health challenge, as diabetic kidney disease (DKD) is increasingly recognized as the primary contributor to both chronic and end-stage renal disease worldwide (^1^). The prevalence of DKD has been increasing in recent years; evidence has shown that approximately 20% of the 400 million individuals with diabetes worldwide suffer from DKD (^2^,^3^). Patients with DKD exhibit an increased risk of cardiovascular disease and increased rates of all-cause morbidity and mortality (^4^). However, the clinical management of DKD has not improved much over the past two decades (^5^). Prevention, early detection, and aggressive treatment of DKD in primary health care settings should be prioritized, given its significant global health burden.

Prior research has suggested that inadequate or excessive sleep can significantly increase the likelihood of developing chronic kidney disease (^6^-^8^). A U-shaped correlation has been found between the sleep duration and the likelihood of renal function impairment and proteinuria in individuals with diabetes (^9^,^10^). Notably, in addition to the average sleep duration, the disparity in sleep durations between weekends and weekdays, known as weekend catch-up sleep (WCS), has an important effect on the occurrence and development of renal disease (^11^). However, it remains uncertain whether there are associations between WCS and the risks of renal function impairment and proteinuria in individuals with diabetes.

In this study, we aimed to evaluate the link between WCS and the risk of DKD by utilizing data from the National Health and Nutrition Examination Survey (NHANES). The correlations between WCS and a reduced estimated glomerular filtration rate (eGFR) and proteinuria were examined.

## SUBJECTS AND METHODS

### Study design and population

This study collected data from 15,560 participants from the NHANES 2017-2020 via a cross-sectional design. The NHANES database only differentiated between sleep durations on weekends and weekdays from 2017 to 2020. The NHANES is a survey that is conducted by the National Center for Health Statistics (NCHS) with the aim of providing health and nutrition statistics on the noninstitutionalized civilian population in the United States. It employs a stratified, multistage probability approach to ensure national representation. The survey was approved by the NCHS Research Ethics Review Board, ensuring that all participants provided informed consent. The nutritional and physical health of participants are assessed through standardized in-home interviews, laboratory tests and physical examinations conducted at mobile examination centers (^12^).

**Figure 1:**
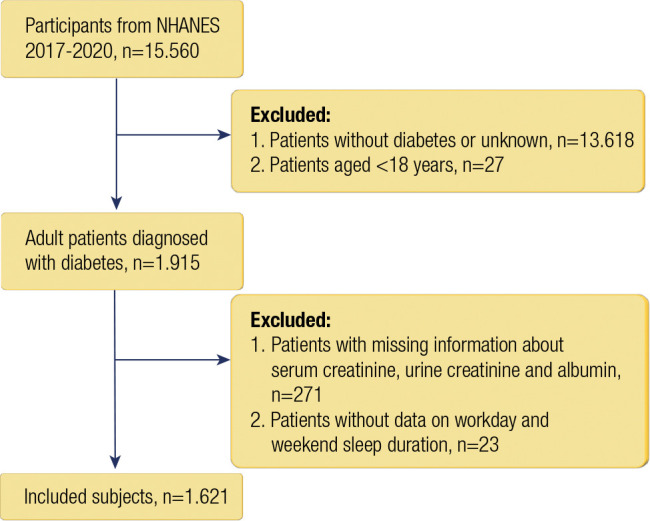
Flowchart of the selection of participants from the NHANES database.

The inclusion criteria for participants in this study were as follows: (^1^) had a diagnosis of diabetes; (^2^) were ≥ 18 years old; and (^3^) had data on serum creatinine, urine creatinine and albumin levels. Participants without workday and weekend sleep duration data were excluded. Finally, 1,621 individuals were included (Figure 1).

### Potential confounding factors

Age (years), sex (male and female), race (Mexican American, non-Hispanic Black, non-Hispanic White, other Hispanic, and other race), education level (less than 9th grade; 9-11th grade, including 12th grade without a diploma; high school grade/general diploma or equivalent; some college or associate of arts degree; and college graduate or above), poverty-to-income ratio (PIR) (<1.3, 1.3-3.5, and ≥3.5), body mass index (BMI) (<25, 25-29.9, and ≥30 kg/m^2^), drinking status (nondrinkers, moderate drinkers, and heavy drinkers), smoking status (never, former and current), physical activity, diabetes-affected eyes/had retinopathy or not, hypertension, dyslipidemia, cardiovascular disease (CVD), hyperuricemia, trouble sleeping, symptoms of obstructive sleep apnea (OSA), and aspartate aminotransferase (AST), alanine aminotransferase (ALT), and high-sensitivity C-reactive protein (hs-CRP) levels were included as potential covariates. Medications, including angiotensin-converting enzyme inhibitors/angiotensin II receptor blockers (ACEIs/ARBs), nephrotoxic drugs, antidiabetic drugs and anxiolytics/sedatives/hypnotics, were also assessed. Individuals who never smoked were determined by their response of “No” to SMQ020 (smoked at least 100 cigarettes in their lifetime). Former smokers were defined as those who responded “Yes” to SMQ020 and “No” to SMQ040 (Do you now smoke cigarettes?). Current smokers were identified on the basis of their response of “Yes” to both SMQ020 and SMQ040. Participants who drank once a week were considered moderate drinkers, and those who drank more than once a week were considered heavy drinkers.

### Main variables

WCS was the main variable and was calculated as the average sleep duration on weekends minus the average sleep duration on weekdays (^13^). In this study, WCS was categorized as < 0 hours, 0-1 hour, 1-2 hours, 2-3 hours, and ≥ 3 hours.

### Outcome variables

DKD was the primary outcome in our study and was defined as a urine albumin/creatinine ratio ≥ 30 mg/g and/or an eGFR < 60 mL/min/1.73 m^2^ (^14^). The secondary outcomes were a reduced eGFR, which was defined as an eGFR < 60 mL/min/1.73 m^2^, and proteinuria, which was characterized by a urine albumin/creatinine ratio ≥ 30 mg/g. eGFR = 141 × min (serum creatinine/κ, 1)^α^ × max (serum creatinine/κ, 1)^-1.029^ × 0.993^Age^ × 1.108 [if female] × 1.159 [if black], where “κ” is 0.7 for females and 0.9 for males, “α” is -0.329 for females and -0.411 for males, “min” indicates the minimum of serum creatinine/κ or 1, and “max” refers to the maximum of serum creatinine/κ or 1.

### Statistical analysis

Continuous variables are presented as the means and standard errors (S.Es), and one-way analysis of variance (ANOVA) was used for comparison among multiple groups. Categorical variables are presented as numbers and percentages [n (%)], and the Rao-Scott chi-square test was employed to compare among groups. The missing values were manipulated, and a sensitivity analysis was conducted (Supplementary Table 1). The sampling weights, including SDMVSTRA, SDMVPSU and WTMECPRP, were applied.

The associations between WCS and DKD, a reduced eGFR or proteinuria were assessed via a weighted multivariate logistic regression model, with adjustment for respective confounding factors. Odds ratios (ORs) and 95% confidence intervals (CIs) were calculated. Additionally, subgroup analyses were conducted for patients of different sexes and those with or without hypertension. The significance level was set at *P* < 0.05. Data extraction, statistical analysis, and the table output were performed via the SAS software 9.4 (SAS Institute, Inc., Cary, NC, USA). The forest plot was drawn in R version 4.2.3 (2023-03-15 ucrt).

## RESULTS

### Demographic and clinical characteristics of the participants

This analysis included 1,621 participants who were diagnosed with diabetes, with an average age of 59.22 years and a sex distribution of 770 (47.10%) females to 851 (52.90%) males. Table 1 displays the demographic and clinical characteristics of the 1,621 participants. A total of 583 (32.83%) participants were diagnosed with DKD in our study. In total, 198 participants had a reduced eGFR, and 499 participants had proteinuria. In addition, the participants were stratified by the duration of WCS, and their characteristics are presented in Table 1. The five groups exhibited significant differences in age, race, CVD status, hyperuricemia status, ALT level, and reduced eGFR (*P* < 0.05).

**Table 1:** Baseline characteristics of adult diabetic patients involved in the study

Variables	Total (n = 1621)	Weekend catch-up sleep, hours				
		<0 (Group 1, n = 237)	0-0.9 (Group 2, n = 855)	1-1.9 (Group 3, n = 226)	2-2.9 (Group 4, n = 139)	≥3 (Group 5, n = 164)
Age, years, Mean (S.E) ^†cdefg^	59.22 (0.69)	59.39 (0.91)	62.53 (0.76)	54.91 (1.38)	53.96 (1.58)	51.69 (1.19)
Gender, n (%)						
Male	851 (52.90)	120 (52.34)	481 (55.22)	108 (53.22)	69 (43.82)	73 (47.69)
Female	770 (47.10)	117 (47.66)	374 (44.78)	118 (46.78)	70 (56.18)	91 (52.31)
Race, n (%) ^†fg^						
Mexican American	227 (9.65)	26 (8.02)	97 (6.95)	36 (12.01)	32 (16.90)	36 (17.56)
Non-Hispanic Black	444 (12.25)	86 (16.10)	187 (9.72)	73 (12.45)	41 (15.37)	57 (17.71)
Non-Hispanic White	497 (58.83)	66 (57.69)	314 (64.41)	60 (57.02)	31 (42.80)	26 (45.35)
Other Hispanic	184 (7.58)	24 (7.64)	94 (6.67)	32 (9.63)	11 (7.03)	23 (9.74)
Other Race	269 (11.68)	35 (10.55)	163 (12.25)	25 (8.89)	24 (17.91)	22 (9.65)
Education level, n (%)						
Less Than 9th Grade	203 (6.82)	20 (4.93)	109 (6.61)	29 (7.63)	20 (8.34)	25 (8.29)
9-11th Grade (Includes 12th grade with no diploma)	202 (9.07)	38 (10.39)	105 (8.93)	22 (6.98)	12 (6.42)	25 (13.36)
High School Grad/GED or Equivalent	409 (31.79)	72 (33.67)	207 (31.96)	54 (31.36)	31 (22.60)	45 (36.13)
Some College or AA degree	506 (30.15)	71 (29.80)	262 (30.15)	72 (27.39)	49 (34.53)	52 (31.54)
College Graduate or above	301 (22.18)	36 (21.21)	172 (22.35)	49 (26.65)	27 (28.11)	17 (10.68)
BMI, n (%)						
<25 kg/m^2^	189 (9.23)	30 (7.19)	105 (10.55)	21 (4.80)	15 (8.27)	18 (12.84)
25-29.9 kg/m^2^	455 (26.40)	70 (29.94)	252 (28.79)	51 (19.65)	38 (25.04)	44 (19.69)
≥30 kg/m^2^	977 (64.37)	137 (62.87)	498 (60.65)	154 (75.55)	86 (66.69)	102 (67.48)
PIR, n (%)						
≤1.3	430 (18.61)	70 (19.46)	222 (18.66)	53 (13.57)	36 (19.00)	49 (24.85)
1.3-3.5	586 (35.66)	87 (31.81)	303 (35.01)	77 (33.93)	57 (44.46)	62 (40.81)
>3.5	398 (35.71)	55 (40.36)	210 (35.11)	73 (43.75)	31 (26.42)	29 (26.52)
Unknown	207 (10.02)	25 (8.36)	120 (11.22)	23 (8.75)	15 (10.12)	24 (7.81)
Smoking status, n (%)						
Never	883 (52.58)	116 (48.94)	436 (48.94)	140 (60.64)	93 (65.19)	98 (55.17)
Former	505 (34.04)	69 (33.31)	305 (37.16)	60 (29.92)	28 (26.47)	43 (30.51)
Current	233 (13.38)	52 (17.74)	114 (13.90)	26 (9.44)	18 (8.34)	23 (14.32)
Drinking status, n (%)						
Non-drinkers	611 (32.87)	93 (35.91)	340 (35.80)	69 (26.35)	49 (27.79)	60 (26.55)
Moderate drinkers	626 (43.19)	95 (41.36)	309 (40.64)	106 (47.58)	53 (46.98)	63 (50.11)
Heavy drinkers	282 (18.80)	34 (18.29)	144 (17.76)	36 (19.32)	33 (23.08)	35 (21.07)
Unknown	102 (5.13)	15 (4.44)	62 (5.80)	15 (6.75)	4 (2.16)	6 (2.27)
Physical activity, met^*^min/week, Mean (S.E)	1200.04 (105.84)	1516.55 (387.60)	1099.20 (91.18)	1349.39 (167.78)	1144.08 (197.42)	1086.08 (225.35)
Diabetic retinopathy, n (%)	231 (13.91)	34 (10.42)	139 (15.63)	22 (12.38)	16 (12.97)	20 (12.81)
Hypertension, n (%)	1348 (82.36)	197 (81.70)	733 (83.99)	184 (80.30)	107 (77.81)	128 (77.13)
Dyslipidemia, n (%) ^†f^	1423 (87.93)	213 (88.80)	770 (90.36)	192 (84.83)	112 (75.97)	141 (87.30)
CVD, n (%) ^†bc^	757 (45.15)	129 (59.39)	426 (46.94)	89 (33.73)	50 (32.64)	67 (42.08)
Hyperuricemia, n (%) ^†gij^	457 (26.63)	68 (26.82)	243 (26.34)	60 (29.93)	45 (36.64)	41 (14.40)
Trouble sleeping, n (%)	595 (40.59)	87 (37.25)	327 (41.04)	79 (44.94)	45 (32.88)	57 (42.42)
Symptoms of OSA, n (%)	978 (60.08)	120 (50.70)	517 (59.26)	152 (70.68)	89 (56.78)	100 (64.52)
Hemoglobin A1c, Mean (S.E)	7.12 (0.06)	7.10 (0.14)	7.11 (0.06)	6.96 (0.14)	7.07 (0.24)	7.59 (0.27)
ALT, U/L, Mean (S.E) ^†^g	25.03 (0.67)	22.98 (1.22)	23.71 (0.73)	27.05 (2.14)	28.90 (2.25)	29.15 (1.54)
AST, U/L, Mean (S.E)	22.37 (0.43)	22.37 (0.69)	22.03 (0.61)	21.41 (0.93)	25.06 (2.43)	23.66 (1.13)
Hs-CRP, mg/L, Mean (S.E)	6.14 (0.39)	6.97 (1.63)	6.30 (0.57)	5.68 (0.52)	5.42 (0.51)	5.34 (0.72)
ACEI/ARB, n (%)	549 (34.65)	80 (33.99)	307 (36.24)	79 (38.75)	40 (26.12)	43 (27.05)
Nephrotoxic drug, n (%)	323 (17.15)	51 (18.23)	181 (17.65)	34 (14.82)	27 (20.68)	30 (13.57)
Anti-diabetic drug, n (%)	1129 (69.48)	169 (64.95)	614 (70.95)	165 (73.77)	77 (57.54)	104 (70.93)
Anxiolytics/sedatives/hypnotics, n (%)	115 (7.56)	14 (4.62)	73 (8.46)	13 (6.76)	4 (3.61)	11 (11.53)
DKD, n (%) ^†fh^	618 (34.70)	94 (32.17)	341 (36.99)	83 (36.53)	41 (20.62)	59 (34.15)
Reduced eGFR, n (%) ^†efg^	261 (13.13)	43 (13.99)	151 (16.36)	30 (8.37)	18 (6.50)	19 (6.81)
Proteinuria, n (%)	499 (27.80)	74 (24.92)	270 (28.34)	70 (32.07)	34 (17.22)	51 (30.88)

Note: GED = general educational development; AA = associate of arts; BMI = body mass index; PIR = poverty-to-income ratio; CVD = cardiovascular disease; OSA = obstructive sleep apnea; AST = aspartate aminotransferase; ALT = alanine aminotransferase; hs-CRP = high-sensitivity C-reactive protein; ACEI = angiotensin converting enzyme inhibitors; ARB = angiotensin II receptor blockers; DKD = diabetic kidney disease; eGFR = estimated glomerular filtration rate.

† Indicate a statistically significant test difference between the two groups [^a^ group 1 *vs*. group 2;

b group 1 *vs*. group 3;

c group 1 *vs*. group 4;

d group 1 *vs*. group 5;

e group 2 *vs*. group 3;

f group 2 *vs*. group 4;

g group 2 vs. group 5;

h group 3 *vs*. group 4;

i group 3 *vs*. group 5; ^j^ group 4 *vs*. group 5]

### Associations of WCS with DKD, a reduced eGFR and proteinuria

Supplementary Table 2 shows the covariates associated with DKD, a reduced eGFR, and proteinuria. The associations of WCS with DKD, a reduced eGFR, and proteinuria can be found in Table 2. In the weighted univariate logistic model (Model 1), when Group 2 (WCS ≥ 0 and < 1 hour) was used as a reference, Group 4 (WCS ≥ 2 and < 3 hours) was associated with lower odds of DKD (OR = 0.48, 95% CI: 0.28-.82, *P* = 0.010), a reduced eGFR (OR = 0.32, 95% CI: 0.12-0.87, *P* = 0.027), and proteinuria (OR = 0.53, 95% CI: 0.28-0.98, *P* = 0.042). After adjusting for age, education level, PIR, smoking status, drinking status, diabetic retinopathy, hypertension, CVD, hyperuricemia, hs-CRP level, and antidiabetic drugs (Model 2), Group 4 (WCS ≥ 2 and < 3 hours) was still related to a lower odds of DKD (OR = 0.51, 95% CI: 0.28-0.93, *P* = 0.030). Similarly, we also found a relationship between Group 4 (WCS ≥ 2 and < 3 hours) and proteinuria risk (Model 2: OR = 0.51, 95% CI: 0.27-0.96, *P* = 0.038) after adjusting for age, race, education level, PIR, smoking status, drinking status, diabetes retinopathy, hypertension, hs-CRP level, and antidiabetic drugs. However, the fully adjusted model did not reveal any significant association of WCS with a reduced eGFR (*P* = 0.083).

**Table 2:** Association of catch-up sleep with DKD, reduced eGFR and proteinuria by multivariate logistic regression model

Weekend catch-up sleep, hours	DKD^#^		Reduced eGFR^*^		Proteinuria^●^	
	OR (95% CI)	*P*	OR (95% CI)	*P*	OR (95% CI)	*P*
0-0.9	Ref		Ref		Ref	
<0	0.76 (0.49-1.20)	0.240	0.66 (0.40-1.07)	0.090	0.88 (0.54-1.42)	0.599
1-1.9	1.16 (0.71-1.90)	0.560	0.74 (0.42-1.30)	0.294	1.27 (0.73-2.23)	0.398
2-2.9	0.45 (0.25-0.79)	0.005	0.35 (0.13-0.90)	0.029	0.51 (0.27-0.97)	0.039
≥3	1.07 (0.66-1.73)	0.789	1.14 (0.43-2.96)	0.795	1.10 (0.70-1.71)	0.684

Note: OR = odds ratio; CI = confidence interval; Ref = reference; DKD = diabetic kidney disease; eGFR = estimated glomerular filtration rate.

# Adjusted for age, race, education level, poverty-to-income ratio (PIR), smoking status, drinking status, diabetes retinopathy, hypertension, cardiovascular disease (CVD), hyperuricemia, and anti-diabetic drug.

^*^ Adjusted for age, race, PIR, smoking status, drinking status, diabetes retinopathy, hypertension, dyslipidemia, CVD, hyperuricemia, alanine aminotransferase and symptoms of obstructive sleep apnea.

● Adjusted for age, race, education level, PIR, smoking status, drinking status, diabetes retinopathy, hypertension, high-sensitivity C-reactive protein, and anti-diabetic drug.

### Subgroup analyses

**Figure 2:**
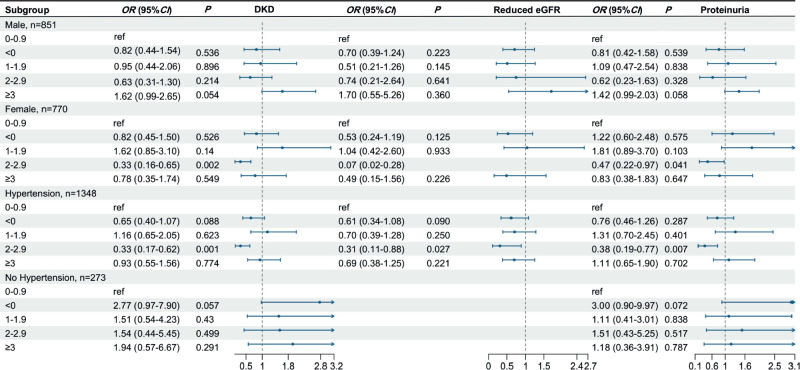
Subgroup analyses stratified by sex and hypertension status.

Figure 2 presents the findings from the subgroup analyses, which were conducted by categorizing participants on the basis of sex and hypertension. The likelihood of developing DKD and proteinuria was significantly lower in female diabetic patients with WCS of ≥ 2 and < 3 hours than in those with WCS of ≥ 0 and <1 hour (OR = 0.40, 95% CI: 0.20-0.78, *P* = 0.010 for DKD; OR = 0.477, 95% CI: 0.22-0.97, *P* = 0.041 for proteinuria). Similarly, diabetic patients who had hypertension and WCS of ≥ 2 and < 3 hours were also found to have a decreased likelihood of developing DKD (OR = 0.39, 95% CI: 0.18-0.81, *P* = 0.012) and proteinuria (OR = 0.38, 95% CI: 0.19-0.77, *P* = 0.007).

## DISCUSSION

The analysis of NHANES 2017-2020 data in this cross-sectional study revealed that individuals with WCS ranging from ≥ 2 to < 3 hours presented a reduced probability of developing DKD and proteinuria after accounting for potential confounding factors. To the best of our knowledge, this study provides initial evidence for an association between WCS and DKD, as well as between WCS and a reduced eGFR and proteinuria, in individuals diagnosed with diabetes.

Adequate sleep is essential for maintaining optimal physical and mental health (^15^). Currently, short or long sleep durations are widely recognized as the most prevalent sleep-related issues (^16^). An increasing amount of evidence suggests a link between the sleep duration and the risk of DKD. A study conducted among Malay and Indian adults with diabetes in Singapore revealed that both abnormally short and long sleep durations were linked to a greater likelihood of developing DKD (^9^). However, the relationships between kidney disease and sleep-related indicators extends beyond this, as several other factors have also been identified. A study in young and middle-aged Japanese individuals with eGFRs ≥ 60 mL/min/1.73 m^2^ revealed that the sleep debt between weekday and weekend sleep durations was related to an elevated risk of proteinuria (^11^). In recent years, the phenomenon of WCS has garnered significant attention (^17^). Multiple studies in the Korean population have established correlations between appropriate WCS patterns and lower risks of dyslipidemia (^18^), metabolic syndrome (^17^,^19^), depression (^20^), and CVD (^21^). However, the evidence on the relationship between WCS and DKD in the American population is still scarce. In the present study, we discovered that the likelihood of developing DKD and proteinuria was significantly lower in diabetic patients who had WCS of ≥ 2 and < 3 hours than in those with WCS of ≥ 0 and < 1 hour. Moreover, according to the results of the subgroup analyses, this connection was consistent in female diabetic patients and in diabetic patients with hypertension. In addition, the present study revealed that the lack of a statistically significant association between WCS and a reduced eGFR may be attributed to the sample source utilized, necessitating future verification. In short, our findings indicate that appropriate sleep supplementation during weekends for individuals with diabetes who experience sleep deprivation throughout the week may mitigate the risk of developing DKD and proteinuria.

According to the currently available research, the mechanisms underlying the link between WCS and DKD in adult patients diagnosed with diabetes are not fully understood. Several potential mechanisms have been proposed as follows: (^1^) An insufficient duration of sleep may exacerbate insulin resistance and increase hypertension risk, both of which are acknowledged as factors contributing to the development of DKD (^22^-^24^). The adequate WCS has the potential to mitigate the occurrence of DKD-associated risk factors. (^2^) A short sleep duration is related to an elevation in proinflammatory markers, which are likely to contribute to the development of DKD (^25^). By modulating the hypothalamic-pituitary axis and sympathetic nervous system, WCS may exert a beneficial effect on activated systemic inflammation (^26^). (^3^) Most renal physiological processes, including sodium excretion, the renin-angiotensin system and blood pressure regulation, exhibit circadian rhythms (^27^). However, a short sleep duration may have a detrimental effect on this chronobiological process and disrupt the circadian rhythm of the kidney, potentially leading to DKD. Adequate restorative sleep may compensate for an insufficient sleep duration and contribute to the restoration of the circadian rhythm of the kidney.

This study had several limitations. First, the cross-sectional design of this study limited the establishment of a causal relationship between WCS and DKD in adult patients diagnosed with diabetes. Second, WCS was determined on the basis of participants’ self-reports of sleep durations during weekdays and weekends rather than being objectively measured via methods such as actigraphy or polysomnography. Therefore, it is possible that the study may have been affected by recall bias. Finally, this study represented only the American population, and the generalizability of our findings to the broader population remains uncertain. In the future, the causal relationship between WCS and DKD should be studied prospectively to improve the understanding of sleep hygiene concepts.

In conclusion, in summary, we found that WCS ≥ 2 and < 3 hours was associated with decreased odds of developing DKD and proteinuria in American adult patients diagnosed with diabetes, particularly among females and patients with hypertension.

## Data Availability

the datasets used and/or analyzed during the current study are publicly available from the NHANES database.

## Appendix

**Supplementary Table 1:** Sensitivity analysis before and after interpolation

Variables	Before imputation	After imputation	P
Education level, n (%)			0.700
Less Than 9th Grade	203 (6.84)	204 (6.84)	
9-11th Grade (Includes 12th grade with no diploma)	201 (9.06)	201 (9.02)	
High School Grad/GED or Equivalent	406 (31.80)	408 (31.75)	
Some College or AA degree	505 (30.24)	509 (30.29)	
College Graduate or above	297 (22.05)	299 (22.10)	
BMI, n (%)			0.730
<25 kg/m^2^	187 (9.28)	189 (9.23)	
25-29.9 kg/m^2^	449 (26.45)	455 (26.40)	
≥30 kg/m^2^	961 (64.27)	977 (64.37)	
Smoking Status, n (%)			0.757
Never	882 (52.58)	883 (52.58)	
Former	504 (34.03)	505 (34.04)	
Current	233 (13.39)	233 (13.38)	
CVD, n (%)			0.127
No	861 (54.94)	864 (54.85)	
Yes	753 (45.06)	757 (45.15)	
Trouble sleeping, n (%)			0.158
No	1024 (59.38)	1026 (59.41)	
Yes	595 (40.62)	595 (40.59)	
Symptoms of OSA, n (%)			0.142
No	643 (39.93)	643 (39.92)	
Yes	976 (60.07)	978 (60.08)	
AST, U/L, Mean (S.E)	22.38 (0.44)	22.37 (0.43)	0.648
Hs-CRP, mg/L, Mean (S.E)	6.17 (0.39)	6.14 (0.39)	0.066

Note: GED=general educational development; AA=associate of arts; BMI=body mass index; CVD=cardiovascular disease; OSA=obstructive sleep apnea; AST=aspartate aminotransferase; hs-CRP= high-sensitivity C-reactive protein.

**Supplementary Table 2:** Screening of confounding factors related to outcome variables

Variables	DKD		Reduced eGFR		Proteinuria	
	OR (95%CI)	*P*	OR (95%CI)	*P*	OR (95%CI)	*P*
Age	1.03 (1.01-1.05)	<0.001	1.12 (1.10-1.14)	<0.001	1.01 (1.01-1.03)	0.047
Gender						
Male	Ref		Ref		Ref	
Female	0.83 (0.60-1.14)	0.234	1.07 (0.70-1.62)	0.756	0.77 (0.56-1.05)	0.096
Race						
Mexican American	Ref		Ref		Ref	
Non-Hispanic Black	1.03 (0.75-1.41)	0.857	3.39 (1.85-6.20)	<0.001	0.82 (0.59-1.14)	0.226
Non-Hispanic White	0.83 (0.57-1.20)	0.306	2.35 (1.32-4.19)	0.005	0.62 (0.42-0.92)	0.019
Other Hispanic	0.66 (0.45-0.97)	0.034	1.48 (0.74-2.97)	0.251	0.67 (0.44-1.04)	0.070
Other Race	0.92 (0.59-1.42)	0.686	1.50 (0.52-4.36)	0.438	0.83 (0.52-1.34)	0.437
Education level						
Less Than 9th Grade	Ref		Ref		Ref	
9-11th Grade (Includes 12th grade with no diploma)	0.85 (0.48-1.49)	0.550	1.39 (0.80-2.42)	0.229	0.68 (0.36-1.27)	0.214
High School Grad/GED or Equivalent	0.70 (0.45-1.06)	0.091	1.11 (0.68-1.81)	0.678	0.53 (0.34-0.82)	0.006
Some College or AA degree	0.63 (0.42-0.96)	0.033	1.15 (0.74-1.79)	0.516	0.53 (0.32-0.88)	0.015
College Graduate or above	0.43 (0.27-0.68)	<0.001	0.87 (0.45-1.71)	0.678	0.41 (0.24-0.70)	0.002
BMI						
<25 kg/m^2^	Ref		Ref		Ref	
25-29.9 kg/m^2^	0.86 (0.48-1.54)	0.592	1.23 (0.64-2.37)	0.522	0.79 (0.47-1.33)	0.358
≥30 kg/m^2^	1.06 (0.63-1.78)	0.821	1.10 (0.66-1.82)	0.717	0.98 (0.57-1.70)	0.945
PIR						
<1.3	Ref		Ref		Ref	
1.3-3.5	1.43 (1.13-1.81)	0.005	1.59 (1.07-2.36)	0.025	1.20 (0.95-1.52)	0.112
≥3.5	0.71 (0.48-1.05)	0.084	1.01 (0.57-1.78)	0.983	0.59 (0.42-0.83)	0.004
Unknown	0.73 (0.44-1.19)	0.193	1.07 (0.50-2.30)	0.853	0.68 (0.45-1.02)	0.061
Smoking status						
Never	Ref		Ref		Ref	
Former	1.64 (1.24-2.17)	0.001	1.69 (1.18-2.43)	0.006	1.46 (1.04-2.05)	0.029
Current	1.01 (0.74-1.38)	0.942	0.71 (0.37-1.38)	0.304	1.07 (0.74-1.55)	0.710
Drinking status						
Non-drinkers	Ref		Ref		Ref	
Moderate drinkers	0.67 (0.47-0.95)	0.028	0.61 (0.40-0.92)	0.020	0.74 (0.54-1.02)	0.063
Heavy drinkers	0.61 (0.40-0.94)	0.026	0.39 (0.26-0.59)	<0.001	0.68 (0.47-0.99)	0.048
Unknown	0.84 (0.44-1.57)	0.564	0.80 (0.39-1.63)	0.525	0.91 (0.46-1.77)	0.763
Physical activity	0.85 (0.70-1.03)	0.096	0.83 (0.53-1.29)	0.385	0.86 (0.73-1.02)	0.082
Diabetes retinopathy						
No	Ref		Ref		Ref	
Yes	1.51 (1.02-2.26)	0.042	1.76 (1.01-3.05)	0.046	1.52 (1.05-2.19)	0.029
Unknown	0.54 (0.37-0.80)	0.003	0.47 (0.23-0.95)	0.036	0.55 (0.37-0.81)	0.004
Hypertension						
No	Ref		Ref		Ref	
Yes	3.43 (2.23-5.27)	<0.001	6.09 (2.06-17.98)	0.002	2.79 (1.88-4.16)	<0.001
Dyslipidemia						
No	Ref		Ref		Ref	
Yes	1.63 (0.81-3.26)	0.161	2.19 (1.11-4.33)	0.026	1.33 (0.67-2.66)	0.403
CVD						
No	Ref		Ref		Ref	
Yes	2.05 (1.27-3.32)	0.005	3.73 (2.18-6.38)	<0.001	1.53 (0.93-2.51)	0.094
Hyperuricemia						
No	Ref		Ref		Ref	
Yes	2.27 (1.50-3.42)	<0.001	5.15 (3.36-7.87)	<0.001	1.46 (0.94-2.27)	0.092
Trouble sleeping						
No	Ref		Ref		Ref	
Yes	0.87 (0.60-1.25)	0.432	1.05 (0.77-1.44)	0.734	0.80 (0.53-1.19)	0.253
Symptoms of OSA						
No	Ref		Ref		Ref	
Yes	0.99 (0.80-1.21)	0.896	0.70 (0.51-0.95)	0.025	1.05 (0.82-1.36)	0.671
ALT	0.97 (0.80-1.19)	0.790	0.37 (0.18-0.75)	0.007	1.07 (0.89-1.29)	0.472
AST	1.00 (0.85-1.19)	0.969	0.72 (0.39-1.32)	0.273	1.03 (0.86-1.22)	0.766
Hs-CRP	1.14 (0.99-1.32)	0.075	1.06 (0.89-1.27)	0.512	1.21 (1.04-1.41)	0.017
ACEI/ARB drug						
No	Ref		Ref		Ref	
Yes	1.22 (0.83-1.78)	0.303	1.13 (0.67-1.90)	0.631	1.16 (0.82-1.64)	0.387
Nephrotoxic drug						
No	Ref		Ref		Ref	
Yes	0.84 (0.58-1.21)	0.327	1.25 (0.74-2.11)	0.382	0.76 (0.52-1.12)	0.160
Anti-diabetic drug						
No	Ref		Ref		Ref	
Yes	1.68 (1.21-2.33)	0.003	1.68 (0.90-3.14)	0.101	1.67 (1.13-2.45)	0.011
Unknown	0.96 (0.53-1.75)	0.887	0.65 (0.28-1.50)	0.296	1.00 (0.51-1.99)	0.990
Anxiolytics/sedatives/hypnotics						
No	Ref		Ref		Ref	
Yes	0.57 (0.29-1.11)	0.094	0.68 (0.30-1.56)	0.349	0.54 (0.23-1.27)	0.150

Note: OR=odds ratio; CI=confidence interval; Ref=Reference; GED=general educational development; AA=associate of arts; BMI=body mass index; PIR=poverty to income ratio; CVD=cardiovascular disease; OSA=obstructive sleep apnea; AST=aspartate aminotransferase; ALT=alanine aminotransferase; hs-CRP=high-sensitivity C-reactive protein; ACEI=angiotensin converting enzyme inhibitors; ARB=angiotensin II receptor blockers; DKD=diabetic kidney disease; eGFR= estimated glomerular filtration rate.
